# P-214. Evaluating Longevity: Assessing the Effects of Nipah Community-Based Health Intervention in Bangladesh

**DOI:** 10.1093/ofid/ofaf695.436

**Published:** 2026-01-11

**Authors:** Tonmoy Sarkar, Utpal K Mondal, Kamal Ibne Amin Chowdhury, Wasik Rahman Aquib, Dewan Rahman, Subyeta Binte Sarwar, Mohammed Ziaur Rahman, Mohammad Enayet Hossain, Nazmun Nahar, Md Saiful Islam, Anika Farzin, Fatema Akther Ema, Shadman Sakib Choudhury, Sharmin Sultana, Sayera Banu, Eric Bergeron, Christina Spiropoulou, Jhon D Klena, Trevor Shoemaker, Joel M Montgomery, Tahmina Shirin, Syed Moinuddin Satter

**Affiliations:** icddr,b International Centre for Diarrhoeal Disease Research, Bangladesh, Dhaka, Dhaka, Bangladesh; Charles Sturt University, New South Wales, New South Wales, Australia; icddr,b, Dhaka, Dhaka, Bangladesh; icddr,b International Centre for Diarrhoeal Disease Research, Bangladesh, Dhaka, Dhaka, Bangladesh; icddr,b, Dhaka, Dhaka, Bangladesh; icddrb, Dhaka, Dhaka, Bangladesh; icddr,b International Centre for Diarrhoeal Disease Research, Bangladesh, Dhaka, Dhaka, Bangladesh; icddr,b International Centre for Diarrhoeal Disease Research, Bangladesh, Dhaka, Dhaka, Bangladesh; University of Kassel, Kassel, Hessen, Germany; UNSW, Sydney, New South Wales, Australia; icddr,b International Centre for Diarrhoeal Disease Research, Bangladesh, Dhaka, Dhaka, Bangladesh; icddr,b, Dhaka, Dhaka, Bangladesh; icddr,b, Dhaka, Dhaka, Bangladesh; Institute of Epidemiology, Disease Control and Research IEDCR, Dhaka, Dhaka, Bangladesh; icddr,b International Centre for Diarrhoeal Disease Research, Bangladesh, Dhaka, Dhaka, Bangladesh; Centers for Disease Control and Prevention, Atlanta, Georgia; US CDC, Atlanta, Georgia; CDC, Sierra Leone, Greater Accra, Ghana; Centers for Disease Control and Prevention CDC, Atlanta, Georgia; Centers for Disease Control and Prevention CDC, Atlanta, Georgia; Institute of Epidemiology, Disease Control and Research IEDCR, Dhaka, Dhaka, Bangladesh; icddr,b International Centre for Diarrhoeal Disease Research, Bangladesh, Dhaka, Dhaka, Bangladesh

## Abstract

**Background:**

In Bangladesh, raw date palm sap consumption identified as a key pathway of Nipah virus (NiV) transmission. Preventing fruit bats' (Pteropus giganteus) from accessing date palm sap could significantly reduce the risk of NiV outbreak. Community-based health interventions in 2008-09 and 2013-14 encouraged sap harvesters (gachhis) to use a skirt like barrier made from bamboo, dhoincha (*Sesbania aculeata*), jute stalks or polythene as a low-cost and practical method for preventing of NiV . This paper explores the long-term adherence to skirt use and potential future direction.

**Methods:**

From March to August 2023, we conducted a cross-sectional study in six villages of two Nipah endemic districts. We identified 145 gachhis and invited them to participate in the survey. Blood samples were collected from both the gachhis and community people to assess the seroprevalence of NiV. Qualitative interviews with purposively selected key persons and gachhis were conducted to explore the drives and challenges related to skirt usage. Quantitative data were analyzed using multivariate regression models, while qualitative data underwent thematic analysis.

**Results:**

In the 2022-23, only 3% of harvesters continued using skirt, a decline from the 73% who reported prior use. Key barriers to sustained use included lack of materials, labor demands, and perceived decline in NiV risk due to fewer local bat sightings. None of the 304 serum samples tested positive for anti-NiV antibodies. Still, 60% of harvesters expressed willingness to use protective measures in future. Willingness was higher among harvesters with larger families (aOR 2.69; 95% CI: 1.06–6.84) and more experience (aOR 5.92; 95% CI: 1.38–25.24). Many (18%) gachhis were opting mesh fabric for sap protection due to practicality and economic benefits.

**Conclusion:**

Despite low long-term adherence, gachhis showed strong willingness to adopt physical barriers. The absence of NiV infection in the community and shifting toward mesh fabric may create a false sense of security, potentially increasing the risk of Nipah outbreaks. Community-driven risk communication to discourage raw date palm sap consumption and strict monitoring of sap collection and trade are crucial to prevent future outbreaks.

**Disclosures:**

All Authors: No reported disclosures

